# The effect of immature adult-born dentate granule cells on hyponeophagial behavior is related to their roles in learning and memory

**DOI:** 10.3389/fnsys.2015.00034

**Published:** 2015-03-06

**Authors:** Wei Deng, Fred H. Gage

**Affiliations:** Laboratory of Genetics, The Salk Institute for Biological StudiesLa Jolla, CA, USA

**Keywords:** adult neurogenesis, hippocampus, learning and memory, anxiety, depression

## Abstract

The neurogenesis hypothesis of depression is based on the correlation between the rate of adult hippocampal neurogenesis and the affective status of rodents. However, studies investigating the role of neurogenesis in the causation of mood regulation have reported inconsistent results. Here, we explored whether the affective state can be affected differentially by adult-born neurons with distinctive physiological characteristics at different maturation stages. We revealed that reducing the immature newborn neuron population had no effect on anxiety- or depression-like behaviors in an array of tests; however, it enhanced hyponeophagia in a novelty suppressed feeding test, but only when the novel environment was drastically different from the home cage. We further demonstrated that reducing the immature newborn neuron population led to delayed habituation to a novel environment and impaired contextual learning. Hence, rather than being directly involved in mood regulation, our studies raise the possibility that adult neurogenesis may influence hyponeophagia through its role in mnemonic processing.

## Introduction

The dentate gyrus of the hippocampus is one of the brain regions that experiences continuous addition of new neurons throughout adulthood (Zhao et al., 2008). Adult-born neurons arising from neural progenitor cells (NPCs) in the subgranular zone differentiate and develop into dentate granule cells (DGCs). The newly born DGCs undergo a lengthy process of maturation before they become indistinguishable from their developmentally born counterparts and are fully integrated into the hippocampal network. Compared to mature DGCs, the immature, adult-born DGCs are more excitable and more plastic (Schmidt-Hieber et al., 2004; Ge et al., 2006, 2007; Gu et al., 2012). During the maturation process, environmental inputs have specific modulatory effects on the immature DGCs that can influence their responses after they mature (Tashiro et al., 2007). Previous genetic and opto-genetic studies have demonstrated that adult-born DGCs at the immature stage play a critical role in learning and memory (Deng et al., 2009; Gu et al., 2012).

The theory for a role of hippocampal neurogenesis in depression was first proposed by Jacobs, van Praag and Gage, mainly based on the opposite effect of stress and serotonin on the level of NPC proliferation (Jacobs et al., 2000). This hypothesis was further supported by the findings that anti-depressant treatments can increase the proliferation of NPCs (Malberg and Duman, 2003; Warner-Schmidt and Duman, 2006), whereas stressful experiences, which are typically associated with corticosterone release (Munck et al., 1984), often result in a decrease in NPC proliferation (Gould et al., 1997; Mirescu and Gould, 2006; Surget et al., 2008). Artificial boosting of corticosterone levels also causes decreased NPC proliferation (Karishma and Herbert, 2002). In studies investigating the casual relationship between neurogenesis and affective regulation, some reports, but not others, suggest that hippocampal neurogenesis is necessary for the efficacy of anti-depressants, depending on the specific rodent behavioral tasks used (Santarelli et al., 2003; David et al., 2009). Because immature, adult-born DGCs with distinct physiological properties are critically involved in learning and memory, we postulated that this population of adult-born DGCs may also make specific contributions to anxiety- and depression-related behaviors.

To test this hypothesis, we used *Nestin-tk* transgenic (tg) mice to reduce adult-born DGC populations at various maturation stages (Deng et al., 2009). Examination of these mice in the novelty suppressed feeding (NSF) test using a large, novel arena revealed that a reduction specifically in the immature, adult-born DGC population aggravated hyponeophagia, an indicator of anxiety- and depression-like behavior. However, similar changes in hyponeophagia were not detected when the testing environment was more similar to the home environment in two different tests. Moreover, reducing the number of immature, adult-born DGCs did not alter the behavior of mice in an array of classic tests for anxiety- and depression-related behaviors. Further analysis demonstrated that the immature, adult-born DGCs were involved in both habituation to a novel environment and learning of a new context, consistent with our previous study showing a key function of immature, adult-born DGCs in learning and memory. Therefore, we propose that it is the learning and memory functions of immature adult-born DGCs that, under certain conditions, mediate behavioral changes in hyponeophagia, a commonly utilized measurement for anxiety- and depression-like behaviors.

## Materials and methods

### Mice and treatments

The *Nestin-tk* tg mice were generated as described previously (Deng et al., 2009). Mice were housed two to five per cage under standard 12-h light/dark cycles with free access to food and water. For all experiments, we used both male and female mice in age-matched litters. Wildtype (wt) littermates were used as controls. Mice used for experiments were backcrossed to C57BL/6 for at least nine generations. BrdU (Sigma) was injected intraperitoneally at 50 mg/kg per day. Ganciclovir (GCV, Invivogen) was injected intraperitoneally at 100 mg/kg per day. GCV treatment for behavioral experiments started when the animals were about 8 weeks old. In all experiments, both tg and wt mice were treated with GCV to control for the side effect of GCV treatment. All experimental procedures were approved by the Institutional Animal Care and Use Committee at The Salk Institute for Biological Studies.

### Immunofluorescence staining

To validate the transient ablation of neurogenesis in *Nestin-tk* tg mice, mice were treated with GCV for 14 days and BrdU was administered during the last 4 days of GCV treatment. Mice were sacrificed at 4 weeks post-GCV treatment by transcardial perfusion with saline followed by 4% paraformaldehyde. Brain sections were prepared according to previously reported procedures and a one-in-twelve series was selected for immunostaining (Deng et al., 2009). The primary antibody rat anti-BrdU (Accurate) was used at 1:200 dilution and the secondary antibody donkey anti-rat conjugated with Cy3 (Jackson ImmunoResearch) was used at 1:250 dilution.

### Novelty-suppressed feeding

The procedure for this test was modified from that described by Santarelli et al. (2003). Mice fasted for 24 h prior to the test. Body weights before and after the fasting were recorded. Mice were then introduced into a brightly lit novel chamber with food (regular rodent chow) in a weighing boat in the center of the chamber (Figure S1A). The novel chamber was made of transparent plexi-glass and measured 43 × 43 × 15 cm (W × L × H). Mice were allowed to feed in the novel chamber for 6 min. Latency to the first bite was recorded as a measure of anxiety-related behavior. Subsequently, mice were returned to their home cage where food was available in a weighing boat, and they fed there for 20 min. The amounts of food consumed in both the novel chamber and the home cage were measured. Total food consumption was used as an indicator of feeding motivation. In the experiment described in **Figure 6A**, a replica of the home cage—without top and bedding—was used as the novel chamber.

### Novelty-induced hypophagia

For the novelty-induced hypophagia (NIH) test, we followed the procedure described by Dulawa and colleagues with some modifications (Dulawa et al., 2004). Mice had free access to both food and water during training and were individually housed for at least 5 days before training. Mice were trained to drink diluted, sweetened condensed milk (SCM, 3:1, water:SCM) for 30 min in their home cage for 3 days (Figure S1B). The latency to drink milk and the amount consumed were recorded as baselines. On the fourth day, mice were introduced to a novel cage that was a clean replica of the home cage except that the bedding and top were removed. There the mice were allowed to drink SCM for 10 min. We chose the modified home cage replica as the novel chamber because a considerable proportion of mice did not drink SCM in a brightly lit open field box. The latency to drink milk and the amount consumed were recorded as measures of anxiety-like behavior.

### Open field test

**Figure 1:**
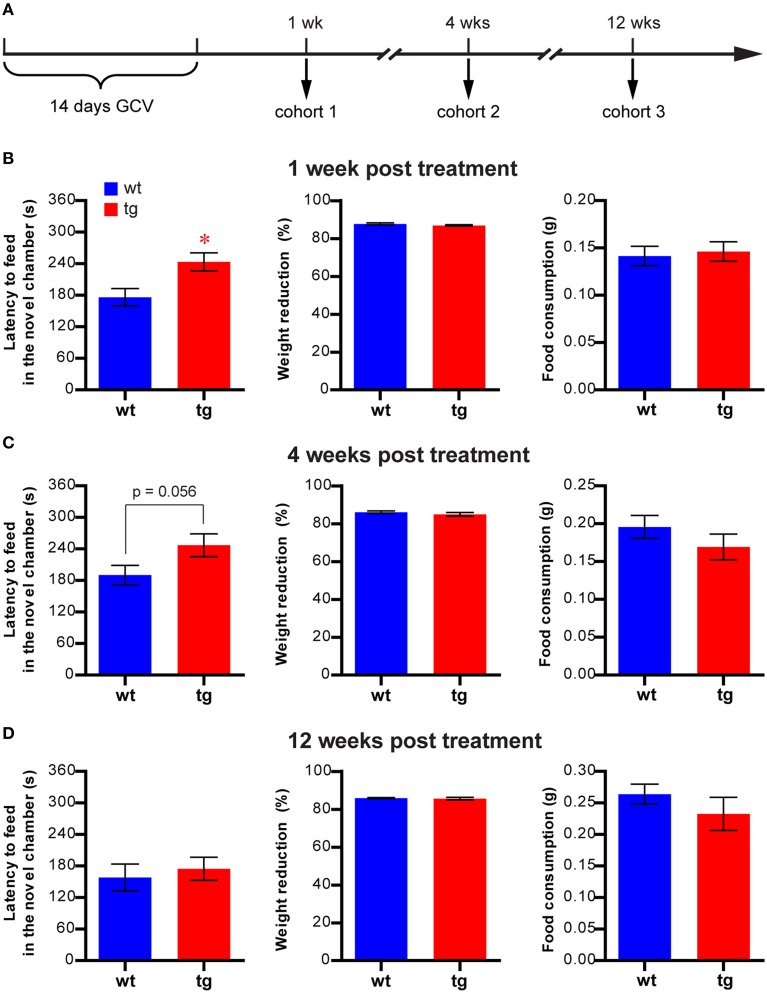
**A reduction in the immature, but not mature, adult-born DGC population prolongs the latency to feed in the NSF. (A)** Experimental design for NSF. **(B)** At 1 week after GCV treatment, latency to feed in the novel chamber is significantly increased in tg mice compared to wt mice [*t*_(51)_ = 2.85, *p* < 0.0063; tg, *n* = 25; wt, *n* = 28]. Reducing neurogenesis in this cohort has no effect on feeding motivation, as indicated by body weight loss [*t*_(51)_ = 1.25, *p* > 0.22] and total food consumption [*t*_(49)_ = 0.32, *p* > 0.74]. **(C)** At 4 weeks after GCV treatment, latency to feed is also increased in tg mice compared to wt mice but does not reach statistical significance [*t*_(25)_ = 2.00, *p* = 0.056; tg, *n* = 13; wt, *n* = 14]. No change in body weight loss [*t*_(25)_ = 1.03, *p* > 0.31] or total food consumption [*t*_(25)_ = 1.17, *p* > 0.25] was detected. **(D)** At 12 weeks after GCV treatment, latency to feed in the novel chamber is not significantly different between tg mice and wt mice [*t*_(28)_ = 0.49, *p* > 0.63; tg, *n* = 15; wt, *n* = 15]. No change in body weight loss [*t*_(28)_ = 0.40, *p* > 0.69] or total food consumption [*t*_(28)_ = 1.03, *p* > 0.31] was detected. Data are shown as mean ± SEM and ^*^ denotes statistically significant difference.

Open field chambers, measuring 43 × 43 × 30 cm (W × L ×H), were obtained from Med Associates Inc. The open field chambers were identical to the novel chambers used in the novelty-suppressed feeding (NSF) (Figure 1 and Figure S1). Two arrays of 16 pulse-modulated infrared beams were installed on both X and Y dimensions to record the movement of the subject. Mice were allowed explore the chamber for 30 min. Their behaviors were recorded and analyzed with the “Activity” software from Med Associates Inc. We designated a square in the center of the area that occupied about a quarter of the total area as the central zone and defined the rest of the area as the periphery zone. We analyzed the total ambulatory distance and total ambulatory time as indications of motility and measured the activity of the subjects and time spent in the central zone as indicators of anxiety-related behavior.

### Light-dark choice

The light-dark choice test was performed in an open field apparatus equipped with a darkened enclosure located along one wall and occupying about one third of the total field. There was an open doorway on the wall of the dark chamber, allowing animals to pass between the darkened enclosure and the lighted compartment. The mice were placed into the dark compartment and were allowed free exploration for 5 min. Their activities were recorded with the “Activity” software. For analysis, the dark zone was defined as 1/4 of the total field covered by the dark chamber, the light zone was defined as the 5/8 of the total field at the opposite end of the dark area and the remaining area was considered the transition zone. Ambulatory distance and total time spent in each zone were quantified.

### Elevated plus maze

The elevated plus maze consisted of a central platform (5 × 5 cm), two closed arms (5 × 30 × 15 cm) and two open arms (5 × 30 cm); it was elevated 30 cm above the floor. A cylinder (diameter = 5 cm) was put on the central platform at the beginning of the test. Mice were placed in the cylinder, which was removed within 1 min. Mice were then allowed to freely explore the maze for 5 min and their behaviors were recorded by a camcorder positioned above the maze. The entries into open and closed arms and time spent on the open arm were scored from the video tapes.

### Forced swimming test

The forced swimming test (FST) was performed in 4-l glass beakers with a diameter of 15 cm containing 15 cm (height) of water (~25°C). A 1-day protocol was used by placing mice into the beakers and recording their behaviors for 6 min using a camcorder (Lutter et al., 2008). To measure depression-like behavior, the duration of immobility in the last 4 min and the latency to the first episode of immobility were scored from video tapes. Immobility was defined as no movements other than those necessary to keep their heads above water.

### Contextual fear conditioning

A contextual fear conditioning protocol that combined the immediate shock procedure with context pre-exposure was used (Fanselow, 1990, 2000). The fear conditioning apparatus and software were obtained from Med Associates, Inc. Mice were handled for at least 1 week before behavioral testing. On the days of testing, mice were acclimated to the procedure room for at least 30 min before testing. On day one, an individual mouse was put into the training chamber (29 × 25 × 26 cm, context A, see Figure S2) and allowed to freely explore for 10 min before being returned to the home cage. In context A, the grid floor of the regular conditioning chamber was covered by a plastic board. We excluded grid floor from pre-exposure because it was the most salient cue for fear response and could elicit a fear response independently of context. On day two, individual mice were subjected to an immediate shock paradigm in context A′, which was identical to context A except with the grid floor exposed (Figure S2). The mouse received a foot shock (0.7 mA, 2 s) 5 s after it was placed into the chamber. Thirty to forty minutes later, mice were individually introduced into context A and context B, which was modified from context A (Figure S2), for 3 min each in a counterbalanced order. Freezing behaviors in context A were analyzed using video freeze software (Med Associates) to evaluate contextual learning.

## Results

### Reducing the population of immature adult-born DGCs but not of their mature counterparts affected hyponeophagia in the NSF test

To reduce the adult-born DGC population at a particular maturation stage, we used the *Nestin-tk* tg mouse model in which proliferating NPCs, which express thymidine kinase (tk) from the *nestin* promoter, can be ablated by the administration of GCV, a nucleotide analog. We showed in our previous study that GCV treatment could effectively reduce the proliferation of NPCs and that neurogenesis could recover after drug withdrawal (Deng et al., 2009). Therefore, by varying the time interval between the GCV treatment and behavioral tests, we were able to study the functions of adult-born DGCs in emotional regulation at various maturation stages. We verified our previous finding that GCV treatment effectively reduced NPC proliferation (Deng et al., 2009) by treating tg mice and wt littermates with GCV for 14 days and sacrificing the mice at 4 weeks post-treatment (Figure S3). BrdU was administered during the last 4 days of GCV treatment to monitor neurogenesis at the time of the drug treatment. The number of BrdU-positive cells was reduced in tg mice, confirming the reduction of neurogenesis by GCV treatment (Figure S3).

To investigate the function of adult-born DGCs in affective regulation, we tested three cohorts of mice in the NSF test at either 1, 4, or 12 weeks after GCV treatment (Figure 1A and Figure S1A). We chose this task because it has been widely used to assess the necessity of adult hippocampal neurogenesis in the efficacy of antidepressants (Santarelli et al., 2003; Petrik et al., 2012). At 1 week after GCV treatment, when the affected adult-born DGCs were about 1–3 weeks of age, the latency to feed in the novel testing chamber, a hyponeophagia measurement commonly used to indicate anxiety level (Santarelli et al., 2003), was significantly longer in tg mice compared to wt controls (Figure 1B). This finding suggests that a reduction in the immature, adult-born DGC population may play a role in anxiety-related behavior. The body weight loss in tg mice post-fasting was similar to that in wt mice, and the tg and wt mice consumed equivalent amounts of food in the testing chamber and their home cages (Figure 1B), suggesting no significant change in feeding motivation in tg mice. Therefore, the enhanced hyponeophagia of the tg mice in the novel chamber was not due to changes in feeding motivation.

In the 4-week post-treatment cohort, the affected adult-born DGCs were about 4–6 weeks of age. In this group, feeding latency was considerably longer in tg mice compared with wt mice, though the difference between the groups did not reach statistical significance (Figure 1C, *p* = 0.056). This result suggested that adult-born DGCs at 4–6 weeks of age could still play a role in hyponeophagia. By contrast, no significant difference could be detected in the feeding latencies between tg mice and wt mice in the 12-week post-treatment cohort (Figure 1D), in which the affected adult-born DGCs had become fully mature. In both the 4- and 12-week cohorts, the weight losses subsequent to fasting and food consumption in home cages were similar between tg and wt mice (Figures 1C,D), suggesting that the feeding motivation was not significantly affected. Hence, enhanced hyponeophagia in NSF was caused by a reduction in the immature, but not mature, adult-born DGC population. Thus, in all the subsequent behavioral experiments, we tested the animals between 1 and 2 weeks post-GCV treatment. Finally, integration of the transgene randomly into mouse genome may cause changes unrelated to neurogenesis alteration and may contribute to behavioral changes. However, the lack of behavioral phenotype in the 12-week post-treatment cohort ruled out this possibility and suggested that the behavioral phenotype was likely due to the changes in neurogenesis.

### Reducing the immature, adult-born DGC population had no effect in the NIH test

**Figure 2:**
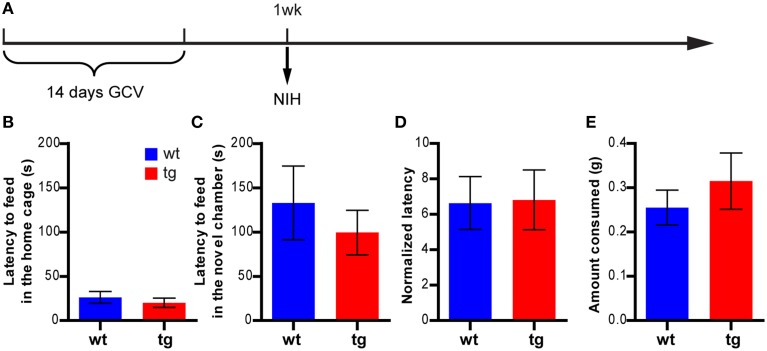
**A reduction in the immature, adult-born DGC population has no effect on feeding behavior in the NIH test. (A)** Experimental design for NIH. **(B)** tg and wt mice are not significantly different in the feeding latency in home cages on the last day of training [*t*_(21)_ = 0.75, *p* > 0.46; tg, *n* = 12; wt, *n* = 11]. **(C)** Reducing neurogenesis does not affect the feeding latency in novel cages [*t*_(21)_ = 0.70, *p* > 0.49]. **(D)** There is no significant difference between tg and wt mice in the normalized feeding latency (feeding latency in novel cages/feeding latency in home cages on the last day of training, [*t*_(21)_ = 0.081, *p* > 0.93]. **(E)** Neurogenesis reduction does not affect the amount of milk consumed [*t*_(21)_ = 0.78, *p* > 0.44]. Data are shown as mean ± SEM.

To confirm these findings, we next examined whether a reduction in the immature, adult-born DGC population could affect anxiety-like behavior in another hyponeophagia-based test, the NIH test (Dulawa and Hen, 2005). In this test, mice were trained to drink sweetened milk in their home cage and were subsequently tested for their behavior in seeking the highly palatable sweetened milk in a novel cage, which was a replica of the home cage but without bedding and top (Figure 2A and Figure S1B). In a cohort of animals tested 1 week after the 14-day GCV treatment, feeding latencies in the novel cages were considerably longer than those in the home cages in both wt and tg mice, as expected (Figures 2B,C). However, to our surprise, no significant difference in feeding latency could be detected between tg and wt mice in either novel cages or home cages (Figures 2B–E). This finding apparently contrasted with what we had observed in the NSF test (Figure 1B). Thus, the behavioral changes in NSF cannot be simply explained by neurogenesis-mediated mood alteration.

### Reducing immature, adult-born DGC population had no effect on anxiety-related behaviors in the open field, the light-dark choice or elevated plus maze tests

Given these results, we further explored the role of immature adult-born DGCs in several classic anxiety and depression tests in which an animal's tendency to avoid risky situations, such as open and brightly lighted environments, was usually measured as an indicator of anxiety status. Because our previous data showed that young newborn DGCs were involved in hyponeophagia (see Figures 1B,C), mice were tested between 1 and 2 weeks after the 14-day GCV treatment for the rest of the study.

**Figure 3:**
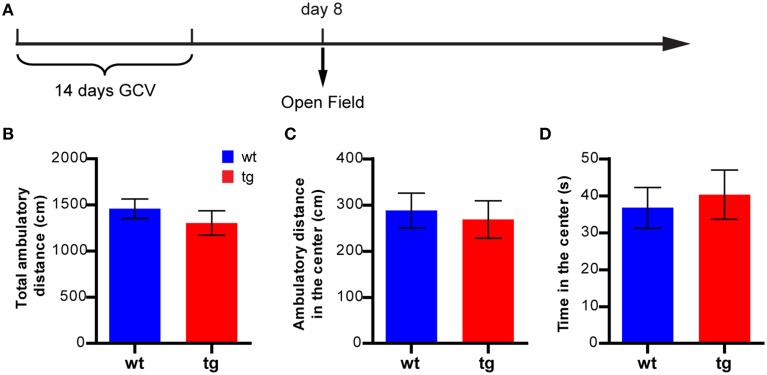
**A reduction in the immature, adult-born DGC population does not affect anxiety-like behavior in the open field test. (A)** Experimental design for the open field test. **(B)** Reducing the number of immature, adult-born DGCs does not affect total ambulatory distance [*t*_(29)_ = 0.91, *p* > 0.37; tg, *n* = 16; wt, *n* = 15]. **(C)** Reducing neurogenesis has no effect on the ambulatory distance in the central zone [*t*_(29)_ = 0.35, *p* > 0.72] or **(D)** time spent in the central zone [*t*_(29)_ = 0.41, *p* > 0.68]. Data are shown as mean ± SEM.

In a new cohort of mice tested at 8 days after GCV treatment in the open field test (Figure 3A), there was no significant difference between tg and wt mice in the ambulatory distance, suggesting no effect on the overall motility of the animals (Figure 3B). Furthermore, there was no significant difference between tg and wt mice in the parameters for anxiety status, such as ambulatory distance and time spent in the center area, suggesting similar anxiety levels (Figures 3C,D). Hence, a reduction in the immature, adult-born DGC population did not result in a change in the anxiety-like behavior in the open field test.

**Figure 4:**
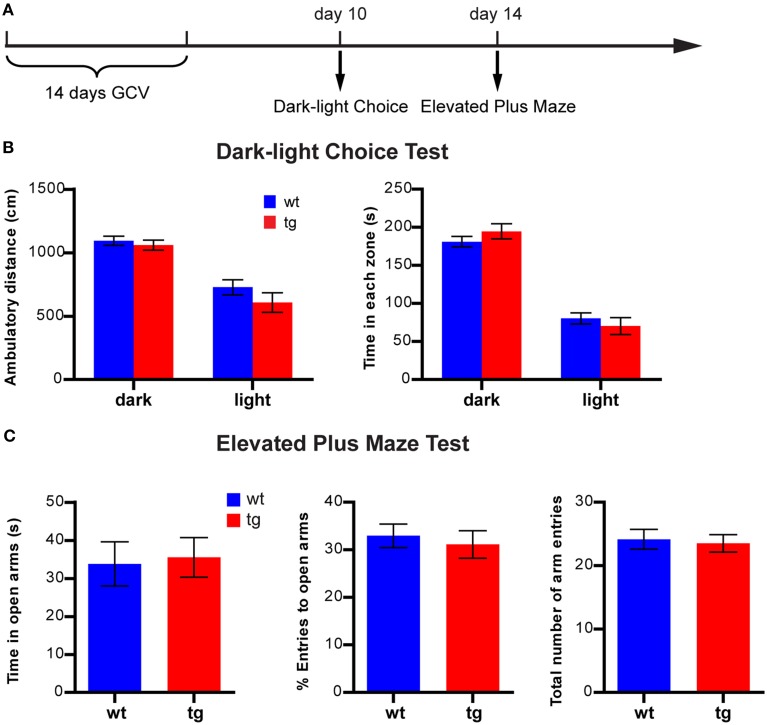
**A reduction in the immature, adult-born DGC population does not affect anxiety-like behavior in the light-dark choice test or the elevated plus maze. (A)** Experimental design for light-dark choice and elevated plus maze. **(B)** In the light-dark choice test, ambulatory distances of both tg and wt mice are longer in the dark compartment than in the lighted compartment, but there is no difference between tg and wt mice [ANOVA, zone × genotype interaction, *F*_(1, 31)_ = 0.47, *p* > 0.49; zone effect, *F*_(1, 31)_ = 42.95, *p* < 0.0001; genotype effect, *F*_(1, 31)_ = 2.86, *p* > 0.10; tg, *n* = 15; wt, *n* = 18]. Both tg mice and wt mice spend more time in the dark compartment than the lighted compartment, but there is no difference between tg and wt mice [ANOVA, zone × genotype interaction, *F*_(1, 31)_ = 1.00, *p* > 0.32; zone effect, *F*_(1, 31)_ = 90.60, *p* < 0.0001; genotype effect, *F*_(1, 31)_ = 0.29, *p* > 0.32]. **(C)** In the elevated plus maze, tg mice and wt mice are not significantly different regarding time spent in the open arm [*t*_(31)_ = 0.22, *p* > 0.82], the percentage of entries into the open arms [*t*_(31)_ = 0.47, *p* > 0.63] and the total number of arm entries [*t*_(31)_ = 0.30, *p* > 0.76]. Data are shown as mean ± SEM.

When another cohort of mice was tested 10 days after treatment in the light-dark choice test (Figure 4A), both tg and wt groups spent less time and showed less activity in the lighted compartment than in the darkened compartment (Figure 4B). In addition, the time spent and activities in the lighted compartments of tg mice were not significantly different from those of the wt controls, suggesting no effect on anxiety state (Figure 4B). Four days later, the same cohort of mice was tested in the elevated plus maze (Figure 4A). No significant difference was detected between tg and wt mice regarding time spent in the open arms, percentage of entries to the open arms and total number of arm entries (Figure 4C). Because the activity/time in the light compartment in the light-dark choice test and the entries/time in the open arms in elevated plus maze were indicators of anxiety-like behaviors, these data suggest that a reduction in the immature adult-born DGC population did not affect anxiety-related behaviors in these tests.

### Reducing the immature, adult-born DGC population had no effect on depression-like behavior in a FST

**Figure 5:**
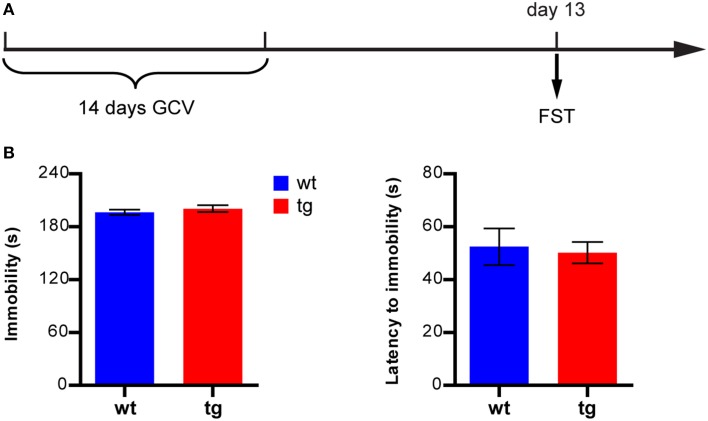
**A reduction in the immature, adult-born DGC population does not affect depression-like behavior in FST. (A)** Experimental design. **(B)** Neither the immobility level nor latency to immobility is significantly different between tg and wt mice [immobility level: *t*_(18)_ = 0.86, *p* > 0.40; latency to immobility: *t*_(18)_ = 0.25, *p* > 0.80, tg, *n* = 8; wt, *n* = 12]. Data are shown as mean ± SEM.

We next asked whether a reduction in the immature adult-born DGC population might affect depression-like behavior in the FST, where immobility is usually measured as an indicator of depression. In a new group of mice subjected to FST 13 days after GCV treatment (Figure 5A), neither the total immobility time nor the latency to the first episode of immobility in tg mice was significantly different from those of wt mice (Figure 5B). Thus, reducing the number of immature adult-born DGCs did not significantly change depression-like behavior in mice in FST.

### Immature, adult-born DGCs mediate hyponeophagia in NSF through their mnemonic function

**Figure 6:**
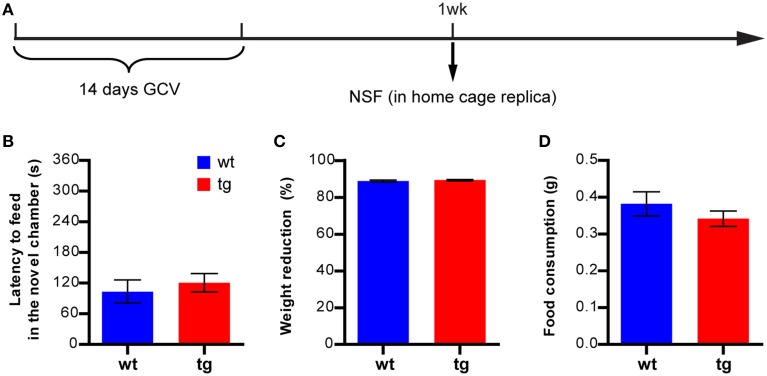
**A reduction in the immature, adult-born DGC population does not affect feeding in a similar environment. (A)** Experimental design. **(B)** Reducing the immature, adult-born DGC population has no effect on the NSF test if the novel feeding occurs in a modified home cage replica, as measured by feeding latency in a novel environment [*t*_(34)_ = 0.60, *p* > 0.55; tg, *n* = 19; wt, *n* = 17]. **(C)** Reducing neurogenesis has no effect on body weight loss [*t*_34)_ = 0.72, *p* > 0.47] or **(D)** total food consumption [*t*_34)_ = 1.07, *p* > 0.29]. Data are shown as mean ± SEM.

In previous studies, we have demonstrated that immature adult-born DGCs are important for learning and memory (Deng et al., 2009). Because a reduction in the immature, adult-born DGC numbers aggravated hyponeophagia in NSF but had no effect in other anxiety- and depression-related tests, the behavioral changes in NSF might result from compromised cognitive ability to learn and adapt to the novel environment rather than from affective changes. To test this possibility, we first attempted to perform the NIH test in the novel chamber used in the NSF test, but found that many mice would not feed in this novel environment that was so completely different from their home cage, possibly due to a lack of motivation. We therefore asked whether the aggravated hyponeophagia could be observed if the novel environment was more similar to home cage. We subjected another cohort of mice to the NSF test at 1 week after GCV treatment, using a modified home cage replica instead of the big, transparent open field chamber as the novel chamber, to reduce the learning demands (Figure 6A). Under this condition, no difference in feeding latency could be detected between tg and wt mice (Figure 6B), suggesting that the enhanced hyponeophagia in the original NSF test (Figure 1B) was likely due to prolonged habituation to a novel environment.

**Figure 7:**
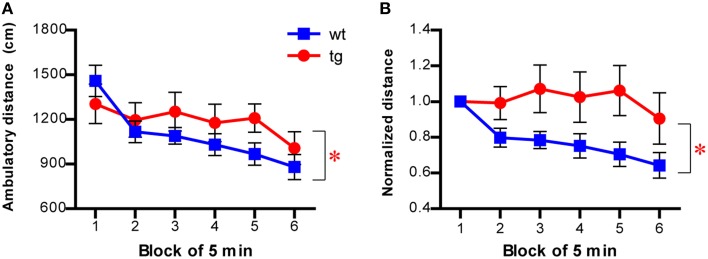
**Habituation to a novel environment is affected in mice with reduced immature adult-born DGCs. (A)** Habituation is slower in tg mice compared to wt mice, as indicated by the ambulatory distance over time [left panel, ANOVA, genotype × time interaction, *F*_(5, 145)_ = 2.53, *p* < 0.032; genotype effect, *F*_(1, 145)_ = 0.68, *p* > 0.42; time effect, *F*_(5, 145)_ = 11.24, *p* < 0.0001; tg, *n* = 16; wt, *n* = 15]. **(B)** The ambulatory activity of mice at every time block is normalized to that of the first block [ANOVA, genotype × time interaction, *F*_(5, 145)_ = 2.70, *p* < 0.024; genotype effect, *F*_(1, 145)_ = 3.82, *p* = 0.060; time effect, *F*_(5, 145)_ = 3.81, *p* < 0.0029]. Data are shown as mean ± SEM and ^*^ denotes statistically significant difference.

Because the dimensions of the novel chamber used in the NSF test were identical to those used in the open field test, we directly examined habituation of mice to the novel chamber in the open field test (described in Figure 3), using motility decline over time as a measure. As expected, there was a significant interaction between group and time in ambulatory distance (Figure 7A). The difference between tg and wt groups was more obvious when the motility of subsequent time blocks was normalized by the motility of the first block, with the activity decreasing more slowly across time in tg mice (Figure 7B). Thus, habituation to a novel environment was affected by a reduction in the immature, adult-born DGC population, suggesting a role for these newborn DGCs in hippocampus-dependent learning. Because we used the same novel chamber in the NSF test (Figure 1 and Figure S1), it is possible that the function of newborn DGCs in learning may influence their feeding behavior, which was used as a measurement for their affective status.

**Figure 8:**
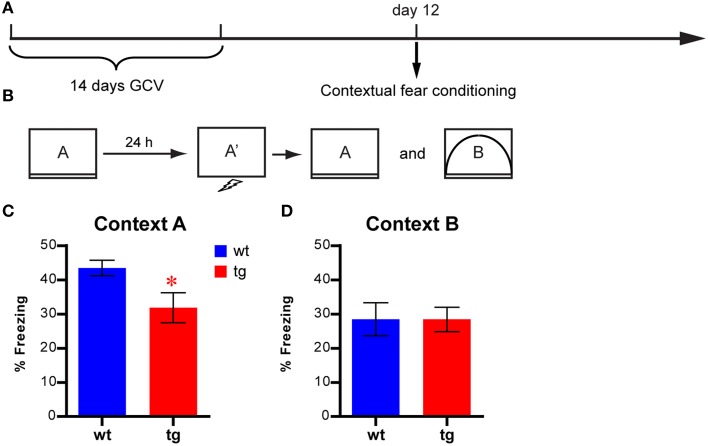
**Contextual learning is affected by reducing the immature, adult-born DGC population (A) Experimental design. (B)** Illustration of the behavioral procedures. **(C)** Freezing behavior is reduced in tg mice compared to wt mice in the conditioned context [i.e., context A, *t*_(17)_ = 2.42, *p* < 0.027; tg, *n* = 9; wt, *n* = 10]. **(D)** No significant difference between tg and wt mice in freezing behavior in a different context [i.e., context B, *t*_(17)_ = 0.0052, *p* > 0.99]. Data are shown as mean ± SEM and ^*^ denotes statistically significant difference.

To directly address whether environmental learning could be affected by a reduction in the immature, adult-born DGC population, we tested a new cohort of mice in a contextual fear conditioning task 12 days after GCV treatment using a protocol combining the contextual pre-exposure with the immediate shock procedure (Figures 8A,B, see Materials and Methods). In this task, contextual learning was thought to occur during the pre-exposure to the novel context under neutral conditions (Rudy et al., 2002). The freezing level displayed by tg mice was significantly lower than that of wt control mice in context A, where the conditioning occurred (Figure 8C), indicating that the tg mice were impaired in learning a new environment. In contrast, we found that reduced immature, adult-born DGC population did not affect freezing behavior in context B, which was different from the conditioning context (Figure 8D). Together, these results suggested that immature, adult-born DGCs were involved in learning and habituation to a novel environment, which could result in altered hyponeophagia under certain circumstances.

## Discussion

In this study, we demonstrated that a reduction in the immature, adult-born DGC population had no effect on the anxiety- and depression-related behaviors of mice in an array of commonly used tasks but did alter hyponeophagia in the NSF test, when the novel testing environment was drastically different from home cages. We further revealed that this behavioral change was likely attributable to a decrement in learning ability rather than to heightened anxiety in mice. It is notable that olfactory neurogenesis was also decreased in GCV-treated *Nestin-tk* mice (Deng et al., 2009). However, because neurogenesis in the olfactory bulb is mainly involved in olfaction-dependent social behavior (Feierstein, 2012), it is unlikely that the affected olfactory neurogenesis is responsible for the observed phenotype here, although this possibility cannot be formally ruled out.

The possibility of a role for adult hippocampal neurogenesis in mood regulation was first proposed by Jacobs and colleagues based on studies about the effect of serotonin neurotransmission and stress on hippocampal neurogenesis in animal models (Jacobs et al., 2000). For instance, anti-depressant treatments, such as selective serotonin reuptake inhibitor (e.g., fluorextine) administration and electroconvulsive shocks, effectively reduced anxiety level and, at the same time, enhanced hippocampal neurogenesis by increasing NPC proliferation (Madsen et al., 2000; Malberg et al., 2000; Nakagawa et al., 2002; Malberg and Duman, 2003; Santarelli et al., 2003; Warner-Schmidt and Duman, 2006; Perera et al., 2007; Petrik et al., 2012). On the other hand, stress, a factor associated with mood disregulations, decreased NPC proliferation (Gould et al., 1992, 1997; Mirescu and Gould, 2006). Elevated levels of stress hormones (e.g., corticosterone) also led to suppression of NPC proliferation (Karishma and Herbert, 2002; David et al., 2009; Surget et al., 2011).

However, recent studies have reported many exceptions to such correlations and revealed a rather complicated relationship between hippocampal neurogenesis and depression. For example, anti-depressant treatment enhanced NPC proliferation in some strains of mice (e.g., 129SvEv, DBA) but not in others (e.g., C56BL/6, balb/c, A/J) (Santarelli et al., 2003; Holick et al., 2008; Miller et al., 2008; David et al., 2009). Furthermore, neurogenesis levels were not always correlated with the behavioral measurements of affective status (Holick et al., 2008; Miller et al., 2008). In mouse models for depression (such as mice with mutations in serotonin receptor 5HT1A or mice treated with unpredictable chronic mild stress or chronic corticosterone), alterations in hippocampal neurogenesis were not consistently observed (Santarelli et al., 2003; Surget et al., 2008; David et al., 2009); neither was an elevation in neurogenesis associated with an antidepressant effect (Fuss et al., 2010; Sahay et al., 2011). Similarly, even though stress is a negative regulator for NPC proliferation, its role in regulating the survival of newly born DGCs is less clear (Wong and Herbert, 2004; Lee et al., 2006; Mirescu and Gould, 2006; Airan et al., 2007; David et al., 2009). Finally, few studies using classical tests for anxiety- and depression- like behaviors reported a direct impact of adult hippocampal neurogenesis on emotional states of animals. In fact, many studies failed to detect any effect of neurogenesis ablation on anxiety- and/or depression-like behaviors in rodent models (Reviewed by Petrik et al., 2012). Consistently, we also found that reducing neurogenesis did not cause behavioral alterations in tests for anxiety- and depression-related behaviors, such as the open field, elevated plus maze, light-dark choice, FST and NIH tests (Figures 2–5). Moreover, most of the classic rodent tests for anxiety- and depression-like behaviors were developed based on their sensitivity to antidepressant medications for human patients. While they served as valuable tools in preclinical research, how the behavioral changes in rodents reflected the mood change was not well understood. Hence, there is no simple causal relationship between neurogenesis and affective regulation.

Although we could not establish a causal relationship between hippocampal neurogenesis and affective regulation, we did find that a reduction in the number of adult-born DGCs at the immature stage enhanced hyponeophagia in the NSF task, a common behavioral paradigm for assessing the efficacy of chronic antidepressants in rodents (Figure 1B). However, enhanced hyponeophagia by neurogenesis reduction was not found in some studies (e.g., Santarelli et al., 2003). The detection of this phenotype is probably due to our specific experimental condition which was associated with high learning demand. Indeed, by altering the novel environment, we showed that this aggravated hyponeophagia could only be detected when the novel chamber was very different from the home cage (Figures 2, 6). In this situation, the measurement of novelty feeding latency could be influenced by how quickly animals could learn and habituate to a novel environment. Our investigation further revealed an involvement of hippocampal neurogenesis in habituation to a novel environment and contextual learning (Figures 7, 8), suggesting that the altered learning ability, rather than heightened anxiety, may account for the increased feeding latency. In agreement with this hypothesis, a role of hippocampal neurogenesis in learning and memory has been established by numerous studies (ref in Deng et al., 2011). Consistent with time frame in detecting the hyponeophagia phenotype, we and others previously reported that immature, adult-born DGCs are involved in learning and memory, possibly due to their distinct physiological characteristics compared to adult-born DGCs (Deng et al., 2009; Gu et al., 2012). Moreover, we were not able to detect the behavioral effect in the NSF task when the population of fully mature, adult-born DGCs was reduced (Figure 1D), a finding that was consistent with a previous report that a reduction in the fully mature, adult-born DGC population had no effect on learning and memory (Deng et al., 2009). Alternatively, the enhanced hyponeophagia in NSF task could be due to elevated stress upon exposure to our particular novel environment. Previous studies suggested that adult neurogenesis can influence anxiety- and depression-like behaviors through regulation of the HPA axis (Surget et al., 2008, 2011), and thus reduction of neurogenesis exacerbated hyponeophagia under stressful conditions (Snyder et al., 2011). However, similar to NSF, the experimental environments used in the open field tests, the light-dark choice and the elevated plus maze were also completely different from the home cage, but no phenotype was detected in these tests by reducing the immature, adult-born DGC population. It was worth noting that the parameters used to indicate anxiety- and depression-like behavior in these tasks were all measured from different compartments in the novel environment. Therefore, the prolonged novelty adaptation could be due to the cognitive impairment caused by a reduction in the immature, adult-born DGC population.

We noticed that our finding of delay habituation to a novel open field chamber was inconsistent with previous reports, which was probably due to the drastic variations in detailed experimental conditions (Meshi et al., 2006; Dupret et al., 2008). For example, these studies used different mice strains, different apparatus configurations, and different methods for data collection and analysis. In addition, these studies also employed different methods to reduce neurogenesis and the developmental stages of the affected adult-born DGCs were not the same among studies. Therefore, it is not possible to direct compare the results among different studies, especially when the overall effect of adult neurogenesis on behavior was small.

The NSF test was also the behavioral paradigm that was consistently used in previous studies to show the importance of hippocampal neurogenesis for the effectiveness of antidepressants (Santarelli et al., 2003; Petrik et al., 2012). However, the involvement of hippocampal neurogenesis in antidepressant efficacy was not consistently detected in other anxiety and depression tests, such as the open field and FST tests. Our findings suggest that the differential dependency in adaptation to novel environments in various anxiety- and depression-related tasks might account for the task-selective detection of the role of hippocampal neurogenesis in anti-depressant efficacy. Moreover, in other studies, hippocampal neurogenesis was only required to mediate the effect of monoaminergic drugs (e.g., fluorextine) but not that of other classes of antidepressants (David et al., 2009). Interestingly, the serotoninergic system, which was the target for the monoaminergic antidepressants, has also been implicated in learning and memory through the serotonin receptors in the septo-hippocampal complex (Buhot et al., 2000). Finally, depression was a common comorbid disorder in patients suffering from dementia and Alzheimer's disease (Doody et al., 2001; Olin et al., 2002). Therefore, modulating serotonin may affect mnemonic processing through altered neurogenesis, which in turn can affect behaviors in the novel and aversive conditions used in many anxiety- and depression-related behavioral tests.

In conclusion, our data indicate that a reduction in the adult-born DGC population at an immature stage did not affect the emotional states of mice but impaired their learning ability, raising the possibility that altered behavior in some anxiety- and depression-related tests with high learning demand may be due to affective cognition.

## Author contributions

WD and FG contributed to study concept and design, interpretation of data and preparation of the manuscript. WD performed all the experiments and data analysis.

### Conflict of interest statement

The authors declare that the research was conducted in the absence of any commercial or financial relationships that could be construed as a potential conflict of interest.

## Supplementary material

Figure S1 **Experimental apparatus and designs for the novelty-suppressed feeding test (A) and the novelty-induced hypophagia test (B)**.

Figure S2 **Illustration of several contexts used in the contextual conditioning test**.

Figure S3 **Reduction of neurogenesis in *Nestin-tk* transgenic mice. (A)** The experimental scheme. *Nestin-tk* (tg) and wildtype control (wt) mice were treated with GCV for 14 days and were injected with BrdU on the last 4 days of GCV treatment to label the proliferating NPCs. Mice were sacrificed 4 weeks later for histological examination of neurogenesis. **(B)** Representative images of BrdU staining in wt and tg mice. **(C)** The number of BrdU-labeled cells is greatly reduced in tg mice [*t*_(18)_ = 5.505, *p* < 0.0001]. The bar represents 100 μm for all panels. Data are shown as mean ± SEM and ^*^ denotes statistically significant difference.

## References

[B1] Airan R. D. Meltzer L. A. Roy M. Gong Y. Chen H. Deisseroth K. (2007). High-speed imaging reveals neurophysiological links to behavior in an animal model of depression. Science 317, 819–823. 10.1126/science.1144400 17615305

[B2] Buhot M. C. Martin S. Segu L. (2000). Role of serotonin in memory impairment. Ann. Med. 32, 210–221. 10.3109/07853890008998828 10821328

[B3] David D. J. Samuels B. A. Rainer Q. Wang J. W. Marsteller D. Mendez I.. (2009). Neurogenesis-dependent and -independent effects of fluoxetine in an animal model of anxiety/depression. Neuron 62, 479–493. 10.1016/j.neuron.2009.04.017 19477151 PMC2759281

[B4] Deng W. Saxe M. D. Gallina I. S. Gage F. H. (2009). Adult-born hippocampal dentate granule cells undergoing maturation modulate learning and memory in the brain. J. Neurosci. 29, 13532–13542. 10.1523/JNEUROSCI.3362-09.2009 19864566 PMC2787190

[B5] Doody R. S. Stevens J. C. Beck C. Dubinsky R. M. Kaye J. A. Gwyther L.. (2001). Practice parameter: management of dementia (an evidence-based review). Report of the Quality Standards Subcommittee of the American Academy of Neurology. Neurology 56, 1154–1166. 10.1212/WNL.56.9.1154 11342679

[B6] Dulawa S. C. Hen R. (2005). Recent advances in animal models of chronic antidepressant effects: the novelty-induced hypophagia test. Neurosci. Biobehav. Rev. 29, 771–783. 10.1016/j.neubiorev.2005.03.017 15890403

[B7] Dulawa S. C. Holick K. A. Gundersen B. Hen R. (2004). Effects of chronic fluoxetine in animal models of anxiety and depression. Neuropsychopharmacology 29, 1321–1330. 10.1038/sj.npp.1300433 15085085

[B8] Dupret D. Revest J. M. Koehl M. Ichas F. De Giorgi F. Costet P.. (2008). Spatial relational memory requires hippocampal adult neurogenesis. PLoS ONE 3:e1959. 10.1371/journal.pone.0001959 18509506 PMC2396793

[B9] Fanselow M. S. (1990). Factors governing one trial contextual conditioning. Anim. Learn. Behav. 18, 264–270 10.3758/BF03205285

[B10] Fanselow M. S. (2000). Contextual fear, gestalt memories, and the hippocampus. Behav. Brain Res. 110, 73–81. 10.1016/S0166-4328(99)00186-2 10802305

[B11] Feierstein C. E. (2012). Linking adult olfactory neurogenesis to social behavior. Front. Neurosci. 6:173. 10.3389/fnins.2012.00173 23226115 PMC3510682

[B12] Fuss J. Ben Abdallah N. M. Vogt M. A. Touma C. Pacifici P. G. Palme R.. (2010). Voluntary exercise induces anxiety-like behavior in adult C57BL/6J mice correlating with hippocampal neurogenesis. Hippocampus 20, 364–376. 10.1002/hipo.20634 19452518

[B13] Ge S. Goh E. L. Sailor K. A. Kitabatake Y. Ming G. L. Song H. (2006). GABA regulates synaptic integration of newly generated neurons in the adult brain. Nature 439, 589–593. 10.1038/nature04404 16341203 PMC1420640

[B14] Ge S. Yang C. H. Hsu K. S. Ming G. L. Song H. (2007). A critical period for enhanced synaptic plasticity in newly generated neurons of the adult brain. Neuron 54, 559–566 10.1016/j.neuron.2007.05.002 17521569 PMC2040308

[B15] Gould E. Cameron H. A. Daniels D. C. Woolley C. S. McEwen B. S. (1992). Adrenal hormones suppress cell division in the adult rat dentate gyrus. J. Neurosci. 12, 3642–3650. 1527603 10.1523/JNEUROSCI.12-09-03642.1992 PMC6575731

[B16] Gould E. McEwen B. S. Tanapat P. Galea L. A. Fuchs E. (1997). Neurogenesis in the dentate gyrus of the adult tree shrew is regulated by psychosocial stress and NMDA receptor activation. J. Neurosci. 17, 2492–2498. 9065509 10.1523/JNEUROSCI.17-07-02492.1997 PMC6573503

[B17] Gu Y. Arruda-Carvalho M. Wang J. Janoschka S. R. Josselyn S. A. Frankland P. W.. (2012). Optical controlling reveals time-dependent roles for adult-born dentate granule cells. Nat. Neurosci. 15, 1700–1706. 10.1038/nn.3260 23143513 PMC3509272

[B18] Holick K. A. Lee D. C. Hen R. Dulawa S. C. (2008). Behavioral effects of chronic fluoxetine in BALB/cJ mice do not require adult hippocampal neurogenesis or the serotonin 1A receptor. Neuropsychopharmacology 33, 406–417. 10.1038/sj.npp.1301399 17429410

[B19] Jacobs B. L. van Praag H. Gage F. H. (2000). Adult brain neurogenesis and psychiatry: a novel theory of depression. Mol. Psychiatry 5, 262–269. 10.1038/sj.mp.4000712 10889528

[B20] Karishma K. K. Herbert J. (2002). Dehydroepiandrosterone (DHEA) stimulates neurogenesis in the hippocampus of the rat, promotes survival of newly formed neurons and prevents corticosterone-induced suppression. Eur. J. Neurosci. 16, 445–453. 10.1046/j.1460-9568.2002.02099.x 12193187

[B21] Lee K. J. Kim S. J. Kim S. W. Choi S. H. Shin Y. C. Park S. H.. (2006). Chronic mild stress decreases survival, but not proliferation, of new-born cells in adult rat hippocampus. Exp. Mol. Med. 38, 44–54. 10.1038/emm.2006.6 16520552

[B22] Lutter M. Sakata I. Osborne-Lawrence S. Rovinsky S. A. Anderson J. G. Jung S.. (2008). The orexigenic hormone ghrelin defends against depressive symptoms of chronic stress. Nat. Neurosci. 11, 752–753. 10.1038/nn.2139 18552842 PMC2765052

[B23] Madsen T. M. Treschow A. Bengzon J. Bolwig T. G. Lindvall O. Tingstrom A. (2000). Increased neurogenesis in a model of electroconvulsive therapy. Biol. Psychiatry 47, 1043–1049. 10.1016/S0006-3223(00)00228-6 10862803

[B24] Malberg J. E. Duman R. S. (2003). Cell proliferation in adult hippocampus is decreased by inescapable stress: reversal by fluoxetine treatment. Neuropsychopharmacology 28, 1562–1571. 10.1038/sj.npp.1300234 12838272

[B25] Malberg J. E. Eisch A. J. Nestler E. J. Duman R. S. (2000). Chronic antidepressant treatment increases neurogenesis in adult rat hippocampus. J. Neurosci. 20, 9104–9110. 11124987 10.1523/JNEUROSCI.20-24-09104.2000 PMC6773038

[B26] Meshi D. Drew M. R. Saxe M. Ansorge M. S. David D. Santarelli L.. (2006). Hippocampal neurogenesis is not required for behavioral effects of environmental enrichment. Nat. Neurosci. 9, 729–731. 10.1038/nn1696 16648847

[B27] Miller B. H. Schultz L. E. Gulati A. Cameron M. D. Pletcher M. T. (2008). Genetic regulation of behavioral and neuronal responses to fluoxetine. Neuropsychopharmacology 33, 1312–1322. 10.1038/sj.npp.1301497 17609676

[B28] Mirescu C. Gould E. (2006). Stress and adult neurogenesis. Hippocampus 16, 233–238. 10.1002/hipo.20155 16411244

[B29] Munck A. Guyre P. M. Holbrook N. J. (1984). Physiological functions of glucocorticoids in stress and their relation to pharmacological actions. Endocr. Rev. 5, 25–44. 6368214 10.1210/edrv-5-1-25

[B30] Nakagawa S. Kim J. E. Lee R. Malberg J. E. Chen J. Steffen C.. (2002). Regulation of neurogenesis in adult mouse hippocampus by cAMP and the cAMP response element-binding protein. J. Neurosci. 22, 3673–3682. 11978843 10.1523/JNEUROSCI.22-09-03673.2002 PMC6758358

[B31] Olin J. T. Katz I. R. Meyers B. S. Schneider L. S. Lebowitz B. D. (2002). Provisional diagnostic criteria for depression of Alzheimer disease: rationale and background. Am. J. Geriatr. Psychiatry 10, 129–141. 10.1097/00019442-200203000-00004 11925274

[B32] Perera T. D. Coplan J. D. Lisanby S. H. Lipira C. M. Arif M. Carpio C.. (2007). Antidepressant-induced neurogenesis in the hippocampus of adult nonhuman primates. J. Neurosci. 27, 4894–4901. 10.1523/JNEUROSCI.0237-07.2007 17475797 PMC6672102

[B33] Petrik D. Lagace D. C. Eisch A. J. (2012). The neurogenesis hypothesis of affective and anxiety disorders: are we mistaking the scaffolding for the building? Neuropharmacology 62, 21–34. 10.1016/j.neuropharm.2011.09.003 21945290 PMC3698048

[B34] Rudy J. W. Barrientos R. M. O'Reilly R. C. (2002). Hippocampal formation supports conditioning to memory of a context. Behav. Neurosci. 116, 530–538. 10.1037/0735-7044.116.4.530 12148921

[B35] Sahay A. Scobie K. N. Hill A. S. O'Carroll C. M. Kheirbek M. A. Burghardt N. S.. (2011). Increasing adult hippocampal neurogenesis is sufficient to improve pattern separation. Nature 472, 466–470. 10.1038/nature09817 21460835 PMC3084370

[B36] Santarelli L. Saxe M. Gross C. Surget A. Battaglia F. Dulawa S.. (2003). Requirement of hippocampal neurogenesis for the behavioral effects of antidepressants. Science 301, 805–809. 10.1126/science.1083328 12907793

[B37] Schmidt-Hieber C. Jonas P. Bischofberger J. (2004). Enhanced synaptic plasticity in newly generated granule cells of the adult hippocampus. Nature 429, 184–187. 10.1038/nature02553 15107864

[B38] Snyder J. S. Soumier A. Brewer M. Pickel J. Cameron H. A. (2011). Adult hippocampal neurogenesis buffers stress responses and depressive behaviour. Nature 476, 458–461. 10.1038/nature10287 21814201 PMC3162077

[B39] Surget A. Saxe M. Leman S. Ibarguen-Vargas Y. Chalon S. Griebel G.. (2008). Drug-dependent requirement of hippocampal neurogenesis in a model of depression and of antidepressant reversal. Biol. Psychiatry 64, 293–301. 10.1016/j.biopsych.2008.02.022 18406399

[B40] Surget A. Tanti A. Leonardo E. D. Laugeray A. Rainer Q. Touma C.. (2011). Antidepressants recruit new neurons to improve stress response regulation. Mol. Psychiatry 16, 1177–1188. 10.1038/mp.2011.48 21537331 PMC3223314

[B41] Tashiro A. Makino H. Gage F. H. (2007). Experience-specific functional modification of the dentate gyrus through adult neurogenesis: a critical period during an immature stage. J. Neurosci. 27, 3252–3259. 10.1523/JNEUROSCI.4941-06.2007 17376985 PMC6672473

[B42] Warner-Schmidt J. L. Duman R. S. (2006). Hippocampal neurogenesis: opposing effects of stress and antidepressant treatment. Hippocampus 16, 239–249. 10.1002/hipo.20156 16425236

[B43] Wong E. Y. Herbert J. (2004). The corticoid environment: a determining factor for neural progenitors' survival in the adult hippocampus. Eur. J. Neurosci. 20, 2491–2498. 10.1111/j.1460-9568.2004.03717.x 15548194 PMC1592224

[B44] Zhao C. Deng W. Gage F. H. (2008). Mechanisms and functional implications of adult neurogenesis. Cell 132, 645–660. 10.1016/j.cell.2008.01.033 18295581

