# Supplementary figures and images for: The effect of immature adult-born dentate granule cells on hyponeophagial behavior is related to their roles in learning and memory
Source: Front Syst Neurosci. 2015 Mar 6;9:34. doi: 10.3389/fnsys.2015.00034 (PMC4351587; doi:10.3389/fnsys.2015.00034)

Figure S1, Deng and Gage

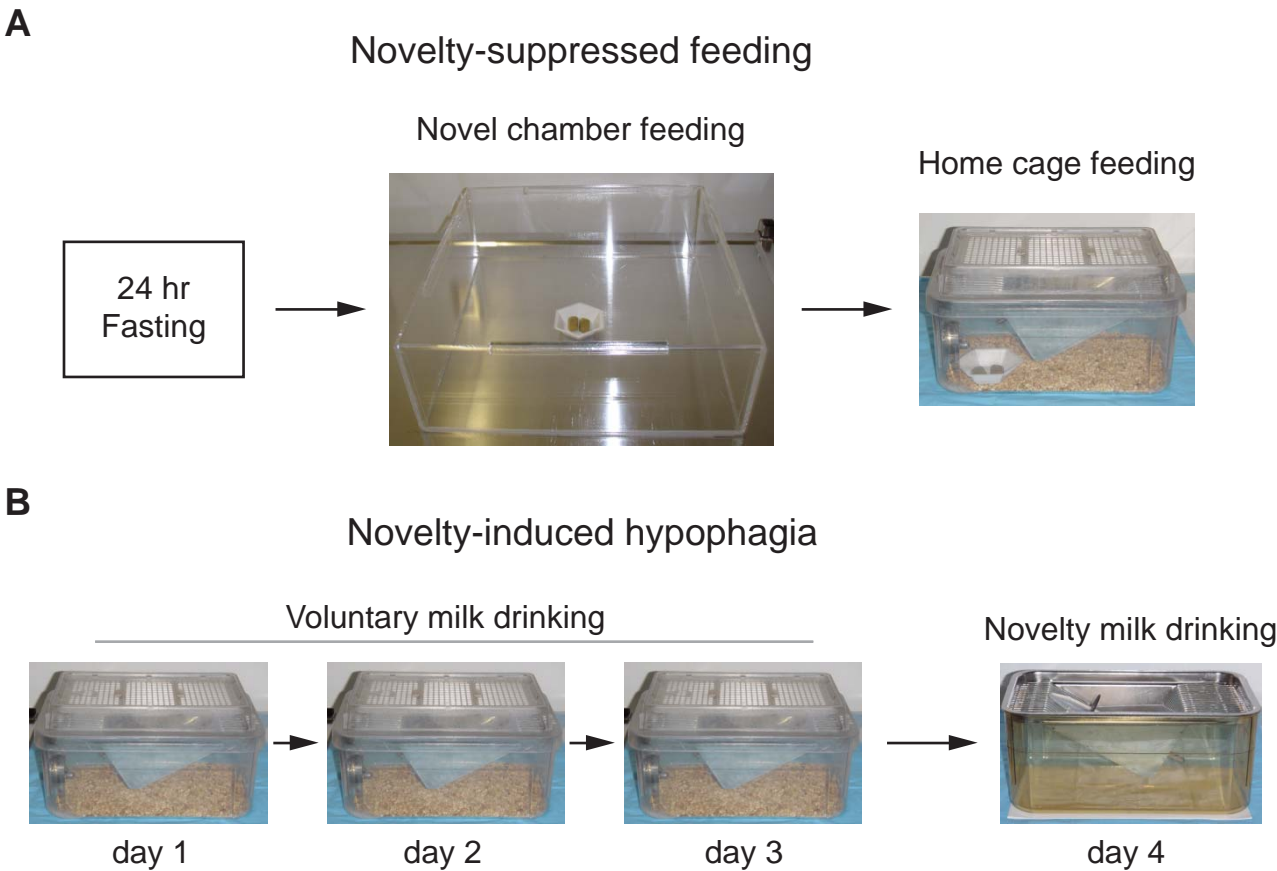

Figure S2, Deng and Gage

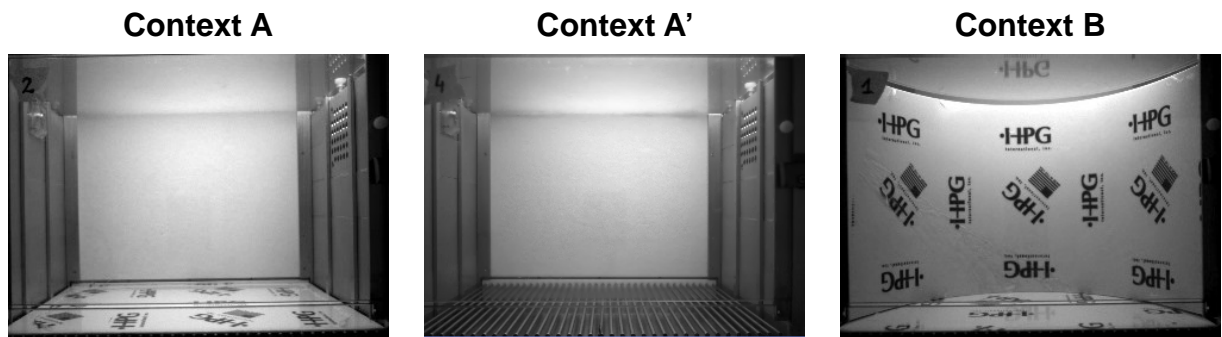

Figure S3, Deng and Gage

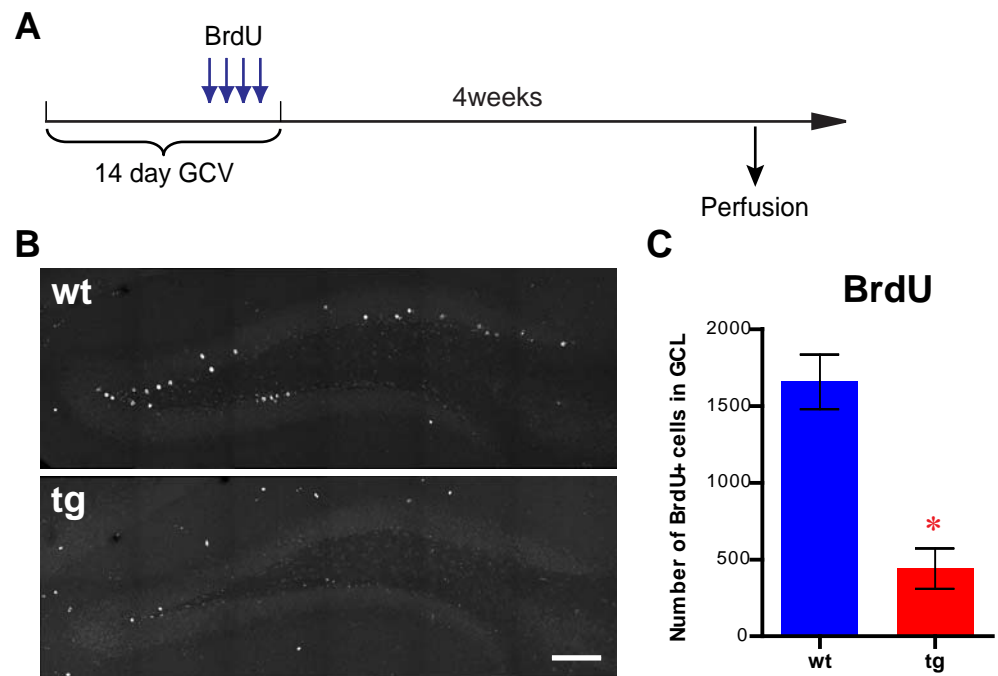

Supplement: Figure S1 — Experimental apparatus and designs for the novelty-suppressed feeding test (A) and the novelty-induced hypophagia test (B). [file Image1.PDF]
